# Systematic Mendelian randomization study of the effect of gut microbiome and plasma metabolome on severe COVID-19

**DOI:** 10.3389/fimmu.2023.1211612

**Published:** 2023-08-16

**Authors:** Han Yan, Si Zhao, Han-Xue Huang, Pan Xie, Xin-He Cai, Yun-Dan Qu, Wei Zhang, Jian-Quan Luo, Longbo Zhang, Xi Li

**Affiliations:** ^1^ Department of Pharmacy, The Second Xiangya Hospital, Central South University, Changsha, Hunan, China; ^2^ Department of Clinical Pharmacology, Xiangya Hospital, Central South University, Changsha, Hunan, China; ^3^ Institute of Clinical Pharmacology, Central South University, Changsha, Hunan, China; ^4^ Hunan Key Laboratory of Pharmacogenetics, Central South University, Changsha, Hunan, China; ^5^ Department of Respiratory Medicine, Xiangya Hospital, Central South University, Changsha, Hunan, China; ^6^ Departments of Neurosurgery, Changde Hospital, Xiangya School of Medicine, Central South University, Changde, Hunan, China; ^7^ Department of Neurosurgery, Xiangya Hospital, Central South University, Changsha, Hunan, China; ^8^ Department of Neurosurgery, Yale School of Medicine, New Haven, CT, United States

**Keywords:** COVID-19, gut microbiome, plasma metabolites, Mendelian randomization analysis, mediation analysis

## Abstract

**Background:**

COVID-19 could develop severe respiratory symptoms in certain infected patients, especially in the patients with immune disorders. Gut microbiome and plasma metabolome act important immunological modulators in the human body and could contribute to the immune responses impacting the progression of COVID-19. However, the causal relationship between specific intestinal bacteria, metabolites and severe COVID-19 remains not clear.

**Methods:**

Based on two-sample Mendelian randomization (MR) framework, the causal effects of 131 intestinal taxa and 452 plasma metabolites on severe COVID-19 were evaluated. Single nucleotide polymorphisms (SNPs) strongly associated with the abundance of intestinal taxa and the concentration of plasma metabolites had been utilized as the instrument variables to infer whether they were causal factors of severe COVID-19. In addition, mediation analysis was conducted to find the potential association between the taxon and metabolite, and further colocalization analysis had been performed to validate the causal relationships.

**Results:**

MR analysis identified 13 taxa and 53 metabolites, which were significantly associated with severe COVID-19 as causal factors. Mediation analysis revealed 11 mediated relationships. Myo-inositol, 2-stearoylglycerophosphocholine, and alpha-glutamyltyrosine, potentially contributed to the association of *Howardella* and *Ruminiclostridium 6* with severe COVID-19, respectively. *Butyrivibrio* and *Ruminococcus gnavus* could mediate the association of myo-inositol and N-acetylalanine, respectively. In addition, *Ruminococcus torques* abundance was colocalized with severe COVID-19 (PP.H4 = 0.77) and the colon expression of permeability related protein RASIP1 (PP.H4 = 0.95).

**Conclusions:**

Our study highlights the potential causal relationships between gut microbiome, plasma metabolome and severe COVID-19, which potentially serve as clinical biomarkers for risk stratification and prognostication and benefit the mechanism mechanistic investigation of severe COVID-19.

## Introduction

Corona Virus Disease 2019 (COVID-19) as a global pandemic continues to spread rapidly across the world causing serious concerns. The individuals with COVID-19 infection could develop fevers, coughing, difficulty in breathing, pneumonia and even death. The prognosis of COVID-19 could be greatly improved with the prevention from the development of severe symptoms. The severity of symptoms varied among the patients which may be attributed to immunity, a combination of basic diseases, The angiotensin-converting enzyme 2 expression and genetic factors (1–5). Nevertheless, the mechanism needs to be further studied.

The gastrointestinal tract is the largest immunological organ in human body and plays an essential role in immunity regulation (6, 7). The microbiome as an important immune regulator in the gastrointestinal system controls host immunity by preserving intestinal mucosa and producing immune regulatory metabolites (such as short chain fatty acids) (8). The intestinal bacteria have been suggested to be closely associated with COVID-19 infection status and severity (9). In the COVID-19 patients, the abundance of *Faecalibacterium prausnitzii*, *Clostridium butyricum* is decreased, while the abundance of *Enterobacter* and *Enterococcus* is increased (10). In addition, the abundance of *Coprobacillus*, *Clostridium ramosum*, and *Clostridium hathewai* in the feces is positively associated with the severity in hospitalized COVID-19 patients, while the abundance of *Faecalibacterium prausnitzii* is negatively correlated (11). Moreover, the severity of COVID-19 has also been reported to be associated with disturbance of various metabolic pathways which are directly or indirectly associated with the systemic inflammatory response observed in patients with severe COVID-19 (12–15). And the metabolites could accurately predict the course of COVID-19, such as tryptophan, kynurenine and 3-hydroxykynurenine, the metabolites of kynurenine pathway (16). In a recent study on immune metabolism, proinflammatory cytokines and chemokines were found to be closely related to metabolites originating in tricarboxylic acid cycle, amino acid metabolism, purine and pyrimidine metabolism and primary bile acid metabolism in severe COVID-19 patients (17). However, most of previous studies are correlational studies, and they are unable to determine whether the selected intestinal bacteria or metabolites has a potential causal relationship with COVID-19 infection and severity. Searching for the causal intestinal bacteria and metabolites of severe COVID-19 could benefit the mechanistic investigation of severe COVID-19 and help the clinical decision before the onset of serious illness.

Causal analysis acts a statistical method allowing the analysis of causal relationship between exposure factors and outcomes. Mendelian randomization (MR) analysis is currently the most commonly used causal inference method in the clinical research frequently using genome-wide association studies (GWAS) summary statistics data. It incorporates germline variants and infers causality based on the principle of random allocation of alleles transferred from parent to offspring at the time of gamete formation, which is less likely to be affected by confounding factors (18). In short, two types of defined phenotypes are studied in MR analysis, one is the exposure factors (candidate causal factors), such as the abundance of intestinal bacteria and another is outcome (affected factors), such as severe COVID-19. Briefly, MR analysis identifies SNPs that are significantly associated with exposure factors at a given p-value threshold (p<1×10^-5^, etc.). Linkage disequilibrium analysis is then applied to select the representative SNPs which can present other SNPs in a high linkage disequilibrium region. These selected SNPs are used as the final instrumental variables (IVs). Based on the IVs, the outcome population is stratified into two groups: the exposure group consisting of individuals who carry the risk alleles of exposures, and the control group consisting of individuals who do not carry the risk alleles. Finally, the differences of outcomes between the two groups are evaluated to infer the causal effects of exposures on outcomes. This causal inference process achieves an effect like that of Randomized Controlled Trials (RCT) and provides a higher level of clinical evidence than correlation analysis (19).

Here, we applied MR analysis to examine the relationship between the gut microbiome, plasma metabolome and severe COVID-19. We found 13 taxa and 53 metabolites act as the causing factors associating with the severity of COVID-19. Moreover, 11 mediated relations among them had been identified. This is the first study to systematically assess the causal relationship between intestinal microbiome, plasma metabolome and the development of severe COVID-19.

## Method and materials

### GWAS datasets and IV selection

This study defined the abundance of intestinal bacteria and the concentration of plasma metabolites as the exposure factors, and severe COVID-19 as the outcome. The genetic association results of the exposure factors were derived from two datasets: the gut microbiome abundance GWAS dataset (MiBioGen consortium, https://mibiogen.gcc.rug.nl/) and the plasma metabolome GWAS dataset (IEU OpenGWAS database (https://gwas.mrcieu.ac.uk/). The gut microbiome abundance GWAS dataset investigated the relationship between whole genome SNPs and the abundance of 211 intestinal taxa in 18473 participants from 24 cohorts in European and American countries (20). Among these taxa, 131 genus and species were selected for the subsequently analysis. The genotyping information was obtained using whole genome genotyping microarrays, and the abundance of each taxon was determined using 16S rDNA sequencing. The plasma metabolome GWAS dataset investigated the relationship between 452 plasma metabolites and germline variants in 7824 European subjects (21). The genetic association results of the outcome were obtained from the COVID-19 Host Genetics Initiative Program (22). This program established a COVID-19 cohort contains 5101 COVID-19 patients with very severe respiratory and 1383241 population controls. The GWAS summary statistics of severe COVID-19 (GCST011075) was downloaded from GWAS Catalog (https://www.ebi.ac.uk/gwas/).

The association p values in each GWAS dataset were used to screen the IVs for each exposure. Due to set different p values as cutoff values would screen different number of IV and then led to different MR results. In our study, four p value levels (p<1×10^-5^, p<1×10^-6^, p<1×10^-7^ and p<1×10^-8^) were set to screen IVs to obtain more information.

### Mendelian randomization and mediation analysis

At first, univariate MR analysis was performed to identify the causal relationship between intestinal bacteria and severe COVID-19 or plasma metabolites and severe COVID-19. According to the number of IV, MR analysis could be divided into two types and different methods are utilized. Polygenic MR analysis was conducted and the inverse variance weighted (IVW) method was employed if the IV number was more than 1, while cis-MR analysis was performed and the Wald ratio model was used if the IV number was equal to 1. For polygenic MR analysis, Cochran Q test and MR-Egger’s intercept were also conducted to investigate the heterogeneity and pleiotropy of the selected IVs. Only the intestinal bacteria and plasma metabolites with MR p<0.05 and without heterogeneity and pleiotropy (het Q>0.05, pleio p>0.05) were included in follow-up analysis.

Mediation analysis is a statistical method that aims to investigate a variable whether mediates the relationship between two other variables. Through mediation MR analysis, we can construct a pathway from exposure factors to outcomes through a mediating factor, which can help elucidate the potential mechanism of exposure factors affecting outcomes. For example, if the abundance of a taxon and the concentration of a metabolite are both causally associated (i.e., positive association) with severe COVID-19, and the abundance of the taxon is also causally associated (i.e., positive association) with the concentration of the metabolite, a triangular relationship is formed. In this relationship, the taxon is the exposure factor, the metabolite is the mediator, and severe COVID-19 is the outcome. To identify the potential mediated relations, bi-directional mediation MR analysis was conducted to identify the potential relationship among the taxa and metabolites that causally associated with severe COVID-19. Firstly, univariate MR analysis was conducted by setting the abundance of selected taxa as the exposures and the concentration of selected metabolites as the outcomes. Then, the analysis was conducted in reverse by setting the concentration of selected metabolites as the exposures and the abundance of selected taxa as the outcomes. To calculate the proportion of mediation effect of the mediator, the following formula was utilized: $\rho_{M}=\frac{\beta_{EM}\times \beta_{MO}}{\beta_{EO}}$ (23, 24). Where $\rho_{M}$ is the proportion of mediation effect of the mediator M, $\beta_{EM}$ is the MR casual effect of exposure E on mediator M, $\beta_{MO}$ is the MR casual effect of mediator M on outcome O, $\beta_{EM}\times \beta_{MO}\;$ represents the ‘indirect’ effect *via* mediator, and $\beta_{EO}$ is the ‘total’ effect of exposure E on outcome O. In addition, due to the mediator may be affected by multiple exposures, to identify the key exposures, multivariate MR is also performed. All of above analysis were conducted by using R package TwoSampleMR (25).

### Database analysis

**Figure 1:**
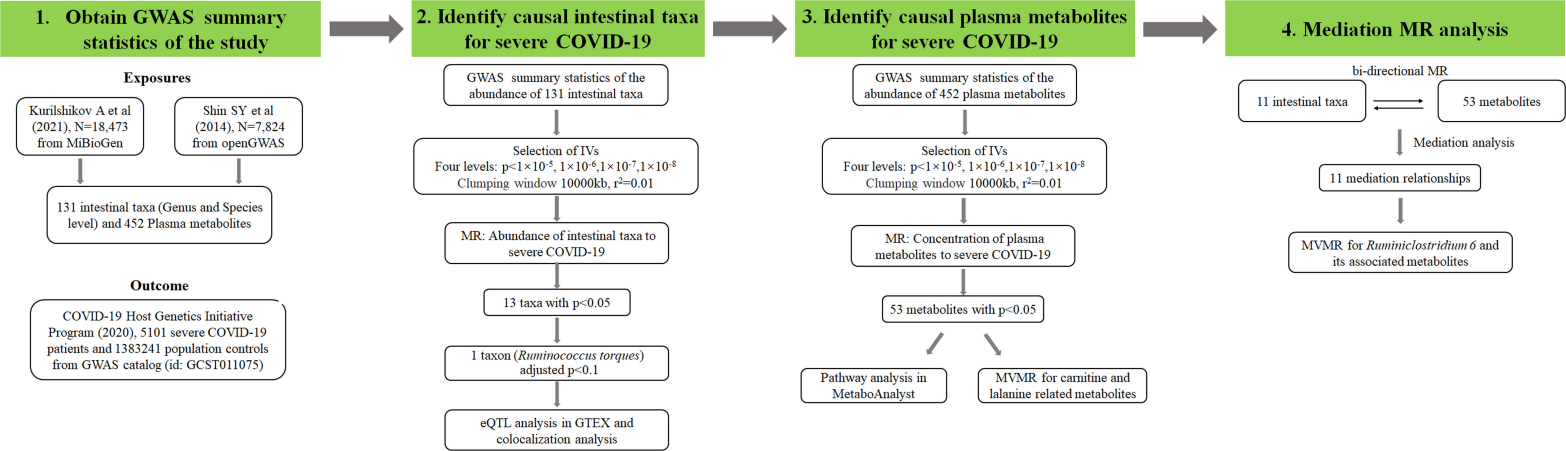
Research flow chart of the study. Exposures means the phenotypes used as the potential causal factors in MR analysis. Outcome means the phenotype used as the affected variable in MR analysis. IVs represents instrument variables which can be screened by different p value levels. N represents the sample size of each dataset. r^2^ was calculated by linkage disequilibrium analysis and adjusted p value was calculated by Bonferroni correction method. MVMR represents multivariate MR.

MetaboAnalyst 5.0 (https://www.metaboanalyst.ca/) was utilized to conduct the enrichment analysis of metabolites. For the most significant exposure in the cis-MR, further colocalization analysis and eQTL analysis were performed. GTEX database (https://gtexportal.org) was used to perform eQTL analysis, and the genes whose expression could be regulated by the IV of candidate intestinal bacteria in colon tissue were screened (26). The colocalization analysis among candidate exposure, severe COVID-19 and the expression of the genes screened by eQTL analysis was also conducted by using R package coloc (27). Web software LocusZoom (http://locuszoom.sph.umich.edu/) was used for regional association plotting, and the flanking size was set as 50kb (28). **Figure 1** showed the research procedures of this study.

## Results

### MR analysis results of gut microbiome

Using univariate MR analysis, we identified 13 taxa significantly associated with severe COVID-19, including *Alloprevotella*, *Anaerotruncus, Bifidobacterium*, *Butyrivibrio*, *Gordonibacter, Howardella, Oxalobacter, Rikenellaceae RC9, Ruminiclostridium 6, Ruminococcus gnavus, Ruminococcus torques, Tyzzerella3 and unknowngenus.id.1000001215* (**Table 1**). Among them, *Anaerotruncus, Butyrivibrio, Gordonibacter, Oxalobacter, Rikenellaceae RC9, Ruminiclostridium 6, Tyzzerella3 and unknowngenus.id.1000001215* showed protective causal effect on severe COVID-19, while *Alloprevotella*, *Bifidobacterium*, *Howardella*, *Ruminococcus torques* and *Ruminococcus gnavus* had positive causal effect. *Bifidobacterium* and *Oxalobacter* showed the same causal effects on COVID-19 at two p value levels.

**Table 1:** Intestinal bacteria significantly causally associated with severe COVID-19.

Taxon	Method	nSNP	OR (95%CI)	p_mr_	Q value	p_pleio_	Level
*Alloprevotella*	IVW	4	1.627 (1.14-2.323)	0.007	0.151	0.489	e5
*Anaerotruncus*	Wald ratio	1	0.213 (0.076-0.595)	0.003	NA	NA	e6
*Bifidobacterium*	IVW	2	2.092 (1.149-3.808)	0.016	NA	NA	e7
*Bifidobacterium*	Wald ratio	1	2.376 (1.211-4.663)	0.012	NA	NA	e8
*Butyrivibrio*	IVW	14	0.83 (0.69-1.000)	0.050	0.052	0.132	e5
*Gordonibacter*	Wald ratio	1	0.578 (0.335-0.998)	0.049	NA	NA	e6
*Howardella*	IVW	7	1.264 (1.009-1.583)	0.042	0.401	0.160	e5
*Oxalobacter*	IVW	11	0.842 (0.709-1.000)	0.050	0.543	0.590	e5
*Oxalobacter*	IVW	4	0.752 (0.578-0.98)	0.035	0.722	0.377	e6
*Rikenellaceae RC9 gutgroup*	Wald ratio	1	0.627 (0.421-0.935)	0.022	NA	NA	e6
*Ruminiclostridium 6*	IVW	14	0.708 (0.544-0.921)	0.010	0.331	0.500	e5
*Ruminococcus gnavus group*	IVW	2	1.703 (1.018-2.849)	0.043	0.538	NA	e6
*Ruminococcus torques group*	Wald ratio	1	6.66 (2.225-19.937)	0.001	NA	NA	e7, e8
*Tyzzerella3*	Wald ratio	1	0.452 (0.255-0.802)	0.007	NA	NA	e8
*unknowngenus.id.1000001215*	IVW	5	0.72 (0.536-0.966)	0.029	0.346	0.336	e5

Note: nSNP, number of selected IVs; OR, odds ratio; CI, confidence interval; p_mr_, MR analysis p value; Q value, Cochran test Q value; p_pleio_, pleiotropy test p value; Level, IVs screen p value level; e5, p<1×10^-5^; e6, p<1×10^-6^; e7, p<1×10^-7^; e8, p<1×10^-8^; NA, not applicable.

### MR analysis results of plasma metabolome

**Figure 2:**
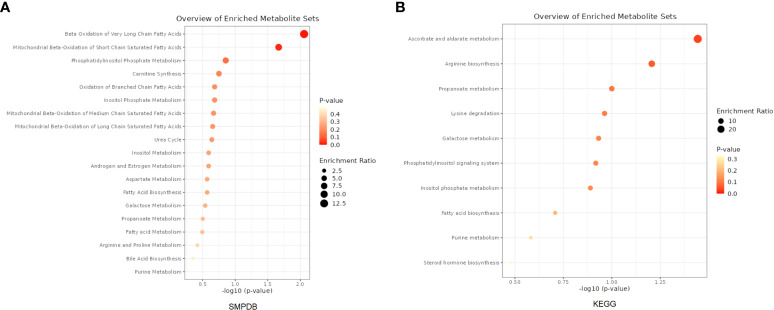
Enrichment analysis results of the causal metabolites of severe COVID-19. **(A)** Based on SMPDB. **(B)** Based on KEGG database.

**Figure 3:**
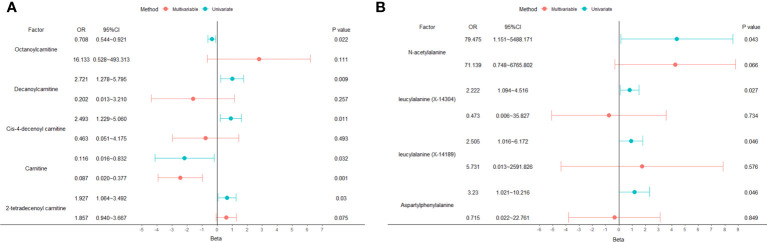
Univariate and multivariate MR analysis results of carnitine and lalanine related metabolites. **(A)** carnitine. **(B)** lalanine.

A total of 53 plasma metabolites had been found significantly associated with severe COVID-19 (**Table 2**). Among them, the plasma concentration of 12-hydroxyeicosatetraenoate, 2-linoleoylglycerophosphocholine, 4-androsten-3beta, 17beta-diol disulfate 1, alpha-glutamyltyrosine, Cis-4-decenoyl carnitine, leucylalanine (X-14304), octanoylcarnitine and urate showed positive causal effect on severe COVID-19 at more than 2 p value levels with the same direction. Enrichment analysis of the 53 identified metabolites indicated that they were significantly enriched in pathways of ascorbate and aldarate metabolism, beta oxidation of very long chain fatty acids and oxidation of branched chain fatty acids (**Figure 2**). 5 identified metabolites were related to carnitine metabolism, include 2-tetradecenoyl carnitine, carnitine, cis-4-decenoyl carnitine, decanoylcarnitine and octanoylcarnitine. After multivariate MR, carnitine (multivariate MR p = 0.001) and 2-tetradecenoyl carnitine (multivariate MR p = 0.075) can be retained in the multivariate MR model (p< 0.1) (**Figure 3A**). In addition, 4 identified metabolites, aspartylphenylalanine, leucylalanine (X-14189), leucylalanine (X-14304) and N-acetylalanine were correlated to lalanine, and only N-acetylalanine (multivariate MR p = 0.066) was retained after multivariate MR (**Figure 3B**).

**Table 2:** Metabolites significantly causally associated with severe COVID-19.

Metabolite	Method	nSNP	OR (95%CI)	p_mr_	Q value	p_pleio_	Level
10-nonadecenoate (19:1n9)	IVW	6	0.242 (0.067-0.878)	0.031	0.707	0.359	e5
12-hydroxyeicosatetraenoate	IVW	8	0.563 (0.374-0.847)	0.006	0.502	0.484	e5
12-hydroxyeicosatetraenoate	IVW	2	0.393 (0.206-0.75)	0.005	0.926	NA	e6
1-docosahexaenoylglycerophosphocholine	IVW	3	0.133 (0.018-0.978)	0.047	0.678	0.603	e5
2-hydroxybutyrate	IVW	3	0.083 (0.008-0.829)	0.034	0.888	0.865	e6
2-linoleoylglycerophosphocholine	IVW	2	27.594 (1.519-501.151)	0.025	0.299	NA	e6
2-linoleoylglycerophosphocholine	Wald ratio	1	132.581 (2.261-7774.598)	0.019	NA	NA	e7, e8
2-stearoylglycerophosphocholine	IVW	10	2.881 (1.108-7.493)	0.03	0.387	0.954	e5
4-androsten-3beta,17beta-diol disulfate 1	IVW	4	0.581 (0.367-0.919)	0.02	0.352	0.366	e6
4-androsten-3beta,17beta-diol disulfate 1	IVW	2	0.513 (0.318-0.825)	0.006	0.597	NA	e7, e8
4-hydroxyhippurate	Wald ratio	1	0.214 (0.052-0.891)	0.034	NA	NA	e6
acetylcarnitine	IVW	3	0.14 (0.023-0.858)	0.034	0.628	0.891	e6
acetylcarnitine	IVW	2	0.1 (0.014-0.7)	0.02	0.817	NA	e7, e8
alpha-glutamyltyrosine	IVW	12	1.941 (1.119-3.367)	0.018	0.816	0.932	e5
alpha-glutamyltyrosine	Wald ratio	1	3.389 (1.022-11.242)	0.046	NA	NA	e6, e7, e8
alpha-hydroxyisovalerate	IVW	8	0.261 (0.102-0.664)	0.005	0.838	0.886	e5
aspartylphenylalanine	Wald ratio	1	3.23 (1.021-10.216)	0.046	NA	NA	e7, e8
carnitine	IVW	9	0.121 (0.017-0.869)	0.036	0.67	0.545	e8
cis-4-decenoyl carnitine	IVW	8	2.461 (1.202-5.038)	0.014	0.952	0.838	e5
cis-4-decenoyl carnitine	IVW	4	2.585 (1.171-5.708)	0.019	0.564	0.985	e6
cis-4-decenoyl carnitine	IVW	2	2.9 (1.221-6.883)	0.016	0.768	NA	e7, e8
citrulline	IVW	4	0.026 (0.001-0.607)	0.023	0.572	0.323	e7
decanoylcarnitine	IVW	3	2.721 (1.278-5.795)	0.009	0.309	0.737	e8
dehydroisoandrosterone sulfate	IVW	2	0.237 (0.071-0.792)	0.019	0.271	NA	e7, e8
gamma-glutamylisoleucine	Wald ratio	1	0.004 (0-0.865)	0.044	NA	NA	e6
gamma-glutamylthreonine	IVW	6	4.644 (1.225-17.604)	0.024	0.456	0.905	e5
hexanoylcarnitine	IVW	2	2.404 (1.094-5.285)	0.029	0.383	NA	e6, e7
hexanoylcarnitine	Wald ratio	1	2.636 (1.168-5.951)	0.02	NA	NA	e8
HWESASXX	IVW	4	3.133 (1.025-9.583)	0.045	0.946	0.884	e5
laurate (12:0)	IVW	9	0.094 (0.009-0.942)	0.044	0.358	0.712	e6
myo-inositol	IVW	26	5.617 (1.86-16.964)	0.002	0.676	0.939	e5
myo-inositol	IVW	5	24.491 (2.129-281.774)	0.01	0.917	0.421	e6
myo-inositol	IVW	2	75.109 (2.544-2217.927)	0.012	0.863	NA	e7
N-acetylalanine	IVW	4	97.063 (2.029-4643.843)	0.02	0.732	0.745	e6
octanoylcarnitine	IVW	12	1.901 (1.094-3.304)	0.023	0.728	0.385	e5
octanoylcarnitine	IVW	6	2.13 (1.177-3.857)	0.013	0.712	0.888	e6
octanoylcarnitine	IVW	3	2.343 (1.253-4.384)	0.008	0.663	0.613	e7
octanoylcarnitine	IVW	2	2.386 (1.251-4.549)	0.008	0.38	NA	e8
pseudouridine	IVW	5	0.005 (0-0.178)	0.003	0.428	0.874	e6
scyllo-inositol	IVW	7	0.44 (0.196-0.988)	0.047	0.851	0.776	e5
taurodeoxycholate	Wald ratio	1	0.435 (0.213-0.891)	0.023	NA	NA	e6
urate	IVW	3	265.964 (2.552-27719.17)	0.019	0.454	0.428	e6
urate	Wald ratio	1	13946.58 (4.065-47843971)	0.022	NA	NA	e7
X-04494	IVW	2	24.449 (1.106-540.287)	0.043	0.277	NA	e6
X-10346	Wald ratio	1	1.928 (1.141-3.257)	0.014	NA	NA	e6
X-11438	IVW	21	1.972 (1.018-3.819)	0.044	0.164	0.914	e5
X-11485	Wald ratio	1	4.45 (1.369-14.469)	0.013	NA	NA	e6
X-11497	IVW	8	7.303 (1.381-38.614)	0.019	0.686	0.637	e5
X-11792	IVW	13	1.363 (1.032-1.799)	0.029	0.509	0.728	e5
X-11792	IVW	5	1.562 (1.075-2.27)	0.019	0.367	0.558	e6
X-11858	Wald ratio	1	2.717 (1.25-5.907)	0.012	NA	NA	e8
X-12013	IVW	3	1.478 (1.058-2.064)	0.022	0.89	0.939	e6
X-12013	Wald ratio	1	1.478 (1.04-2.101)	0.029	NA	NA	e7
X-12188	IVW	7	1.205 (1.012-1.434)	0.036	0.809	0.684	e6
X-12244	IVW	14	3.536 (1.008-12.408)	0.049	0.284	0.048	e5
X-12442	Wald ratio	1	3.566 (1.04-12.224)	0.043	NA	NA	e6, e7, e8
X-12850	Wald ratio	1	0.308 (0.096-0.985)	0.047	NA	NA	e8
X-13477	IVW	5	6.383 (1.082-37.654)	0.041	0.347	0.887	e5
X-13553	IVW	8	0.231 (0.055-0.976)	0.046	0.036	0.075	e5
X-14056	IVW	6	0.244 (0.085-0.702)	0.009	0.665	0.532	e5
X-14086	IVW	11	2.321 (1.218-4.422)	0.01	0.639	0.271	e5
X-14086	IVW	3	3.11 (1.061-9.115)	0.039	0.498	0.984	e6
X-14086	Wald ratio	1	4.736 (1.028-21.819)	0.046	NA	NA	e7, e8
X-14189	Wald ratio	1	2.505 (1.016-6.172)	0.046	NA	NA	e6, e7, e8
X-14208	Wald ratio	1	3.905 (1.309-11.649)	0.015	NA	NA	e8
X-14304	Wald ratio	1	2.741 (1.018-7.38)	0.046	NA	NA	e8
X-18601	IVW	2	0.22 (0.069-0.696)	0.01	0.823	NA	e8

Note: nSNP, number of selected IVs; OR, odds ratio; CI, confidence interval; p_mr_, MR analysis p value; Q value, Cochran test Q value; p_pleio_, pleiotropy test p value; Level, IVs screen p value level; e5, p<1×10^-5^; e6, p<1×10^-6^; e7, p<1×10^-7^; e8, p<1×10^-8^; NA, not applicable.

### Bi-directional mediation analysis results

**Figure 4:**
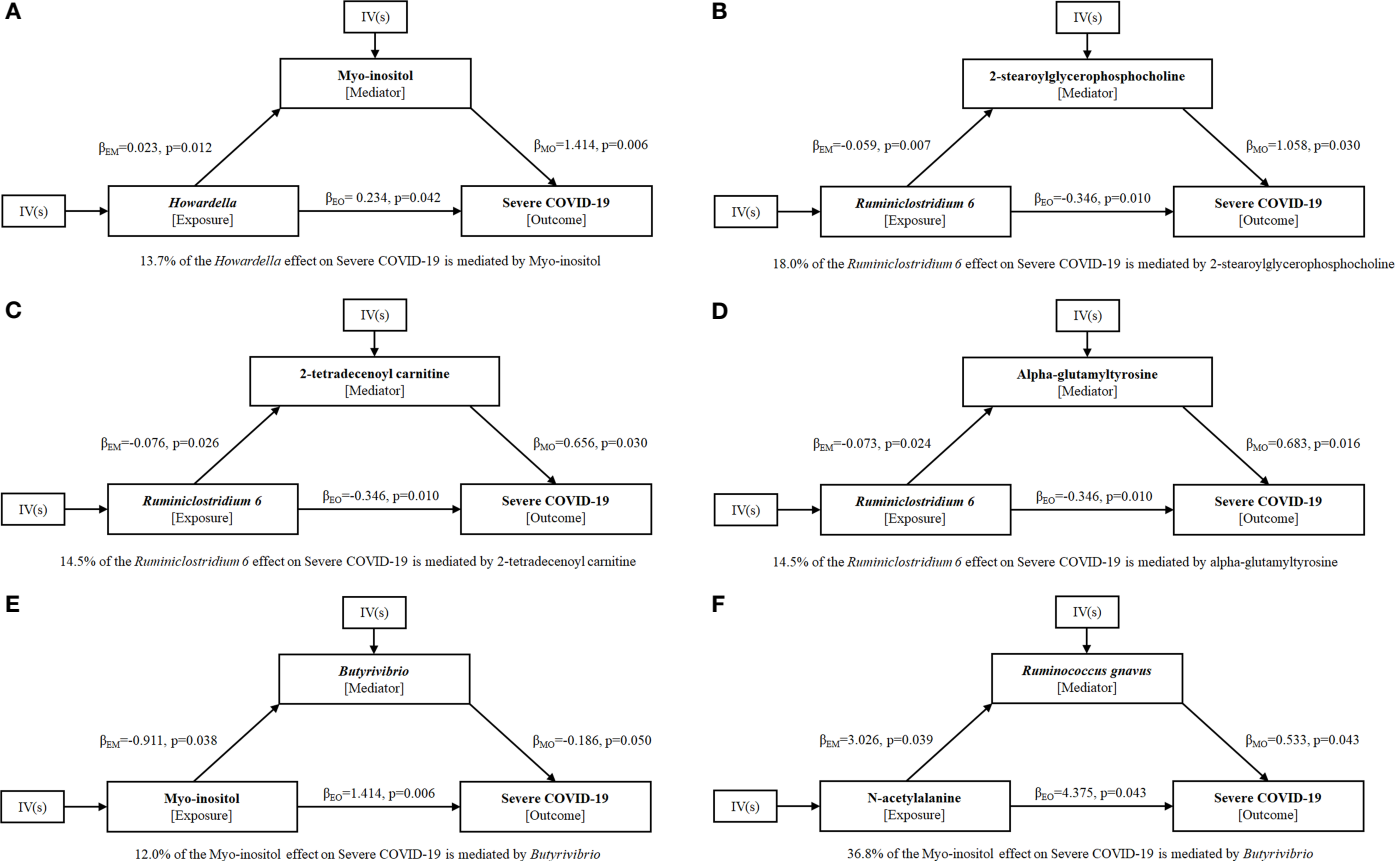
Mediation effect relationship of known metabolites and bacteria on severe COVID-19. $\beta_{EM}$ is the MR casual effect of exposure E on mediator M, $\beta_{MO}$ is the MR casual effect of mediator M on outcome O, and $\beta_{EO}$ is the ‘total’ effect of exposure E on outcome O. The proportion of mediation effect is expressed as a percentage. The p values were calculated by IVW method. **(A)** Howardella, **(B–D)** Ruminiclostridium 6, **(E)** Myo-inositol, **(F)** N-acetylalanine.

To explore the potential mechanism of intestinal microbiome and plasma metabolome in the development of severe COVID-19, bi-directional mediation analysis between intestinal bacteria and plasma metabolites were performed. This analysis was focused on the bacteria and metabolites that significantly causal associated with severe COVID-19 in polygenic MR analysis. A total of 11 mediation relationships were identified (**Table S1**), and 6 of them were composed by known bacteria and metabolites (**Figure 4**). The indirect effect of *Howardella via* myo-inositol was 13.7% (**Figure 4A**). The proportion of mediation effect of *Ruminiclostridium 6* on severe COVID-19 *via* 2-stearoylglycerophosphocholine, 2-tetradecenoyl carnitine, alpha-glutamyltyrosine and X-11497 were 18.0%, 14.5%, 14.5% and 16.7%, respectively (**Figures 4B–D**). *Butyrivibrio* mediated 12% effect of myo-inositol on severe COVID-19 (**Figure 4E**). *Ruminococcus gnavus* mediated more than one third effect (36.8%) of N-acetylalanine (**Figure 4F**). Due to the effects of *Ruminiclostridium 6* on severe COVID-19 was mediated by 4 plasma metabolites, multivariate MR was also performed to find the key metabolites. The multivariate MR results indicated that alpha-glutamyltyrosine (multivariate MR p = 0.027) and 2-stearoylglycerophosphocholine (multivariate MR p = 0.051) were more import than the others (**Table 3**). After adjusting for 2-stearoylglycerophosphocholine, alpha-glutamyltyrosine or both, the effect of *Ruminiclostridium 6* on severe COVID-19 decreased, and the MR p value became non-significant, which suggested that these two metabolites mediate the main effect of *Ruminiclostridium 6* on severe COVID-19 (**Table 4**).

**Table 3:** Multivariate Mendel randomization analysis of *Ruminiclostridium 6* related metabolites.

Exposure	Univariate				Multivariate			
	Beta	SE	OR (95%CI)	p	Beta	SE	OR (95%CI)	p
X-11497	1.896	0.869	6.66 (1.213~36.570)	0.029	0.492	0.806	1.635 (0.337~7.939)	0.542
2-stearoylglycerophosphocholine	1.058	0.488	2.881 (1.107~7.497)	0.030	0.982	0.503	2.671(0.996~7.155)	0.051
2-tetradecenoyl carnitine	0.656	0.303	1.927 (1.064~3.490)	0.030	0.371	0.318	1.450 (0.777~2.703)	0.243
Alpha-glutamyltyrosine	0.683	0.283	1.98 (1.137~3.448)	0.016	0.607	0.274	1.834 (1.072~3.139)	0.027

Note: Beta, effect value calculated by MR analysis; SE, standard error; OR, odds ratio; CI, confidence interval.

**Table 4:** Univariate and multivariate Mendel randomization analysis of *Ruminiclostridium 6*.

Status	Beta	SE	OR (95%CI)	p
Univariate	-0.346	0.135	0.708 (0.544~0.921)	0.010
Adjusted by 2-stearoylglycerophosphocholine	-0.277	0.194	0.758 (0.519~1.109)	0.154
Adjusted by alpha-glutamyltyrosine	-0.320	0.164	0.726 (0.526~1.002)	0.051
Adjusted by both	-0.241	0.167	0.786 (0.567~1.089)	0.148

Note: Beta, effect value calculated by MR analysis; SE, standard error; OR, odds ratio; CI, confidence interval.

#### eQTL and colocalization analysis

**Figure 5:**
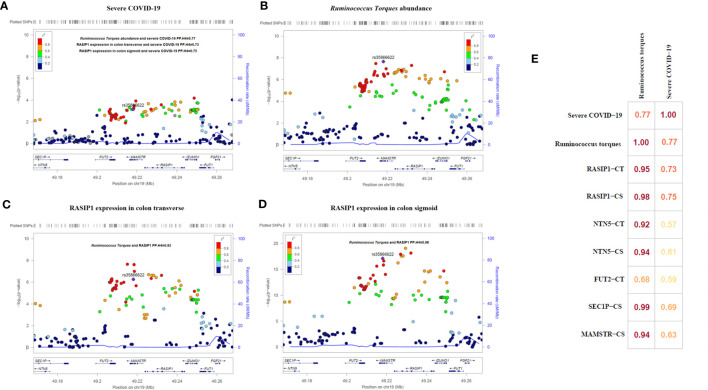
Regional association plots and matrix diagram of colocalization analysis results. In **(A–D)** each point represents an SNP. The abscissa represents the physical position on the chromosome and the vertical axis represents the -log p value. The p values for each SNP were calculated by association analysis. **(E)** Colocalization matrix diagram for Ruminococcus torques, severe COVID-19 and rs35866622 related genes.

After applied multiple test correction based on Bonferroni correction method for cis-MR results, only *Ruminococcus torques* had a trend of positive causal relationship with the severe COVID-19 (Bonferroni adjusted p=0.092, raw p=7.0×10^-4^ [OR=6.66, 95%CI:2.23-19.94]). Using GTEX database, we performed the eQTL analysis and identified rs35866622 (the IV of *Ruminococcus torques*) as a eQTL of RASIP1, NTN5, MAMSTR, SEC1P, IZUMO1, FAM83E, SPHK2 and FUT2. Colocalization analysis revealed that the abundance of *Ruminococcus torques* was highly colocalized with severe COVID-19 (PP.H4 = 0.77, **Figure 5**). Moreover, the abundance of *Ruminococcus torques* was also found to be significantly colocalized with the mRNA expressions of RASIP1, NTN5, MAMSTR, and SEC1P (PP.H4>0.92, **Figure 5**), which indicates that they might be affected by the same cause. Further analysis revealed that only RASIP1 expression had a higher colocalization probability with severe COVID-19. The PP.H4 value in transverse colon and sigmoid colon were 0.73 and 0.75, respectively (**Figure 5**). These findings indicated that RASIP1 expression in colon influenced the risk of respiratory severity in COVID-19 patients.

## Discussion

In this study, the causal relationships of gut microbiome and plasma metabolome for the severity of COVID-19 were investigated using MR analysis. We found that 13 taxa (*Butyrivibrio*, *Howardella*, *Oxalobacter*, *Ruminiclostridium 6*, *Ruminococcus torques*, etc.) and 53 metabolites (2-stearoylglycerophosphocholine, alpha-glutamyltyrosine, carnitine, myo-inositol, etc.) had a potential causal relationship with severe COVID-19. Pathway analysis of the 53 identified metabolites suggested that they were significantly enriched in pathways of ascorbate and aldarate metabolism, beta oxidation of very long chain fatty acids and oxidation of branched chain fatty acids. Mediation analysis among the identified exposures found that the associations of *Howardella, Ruminiclostridium 6*, myo-inositol and N-acetylalanine with severe COVID-19 were likely to be mediated by one or more exposures. After multiple testing correction of cis-MR results, only *Ruminococcus torques* had a trend of positive causal relationship with the severe COVID-19. The increased abundance of *Ruminococcus torques* could be a contributor to the incidence of severe respiratory symptoms in COVID-19 patients. The results of the colocalization analysis revealed that the abundance of *Ruminococcus torques* and the expression of RASIP1 in colon tissue shared a causal factor and had a high colocalization probability with the occurrence of severe respiratory symptoms, implying that they both played important roles in the development of severe COVID-19.

Myo-inositol has been reported to downregulate the expression of IL-6 levels inhibiting the downstream inflammatory response (29). Furthermore, myo-inositol, as precursor of inositol-phosphate, stimulates surfactant production in lung tissue, and thus could represent a potential preventive strategy for COVID-19 (29, 30). Consistent with this, we provided causal evidence for directionally consistent effects of myo-inositol on severe COVID-19. Bi-directional Mediation analysis results indicated that myo-inositol mediated 13.7% effect of *Howardella* on severe COVID-19, while the mediation effect of myo-inositol *via Butyrivibrio* was 12% for severe COVID-19.

A recent study indicates that gut microbiome of patients with post-acute COVID-19 syndrome are characterized by higher levels of *Ruminococcus gnavus (* 31), which has been shown to promote inflammatory responses and impair barrier functions by producing inflammatory polysaccharides (32). We also showed that *Ruminococcus gnavus* causally increases the risk of severe COVID-19 using univariate MR analysis. Furthermore, Mediation analysis results revealed that *Ruminococcus gnavus* mediates more than one third effect (36.8%) of N-acetylalanine on severe COVID-19.

Notedly, *Ruminiclostridium 6* was previously found to have a strong positive correlation with the levels of ghrelin (33), which exerts immunomodulatory functions in COVID-19 infection, such as the suppressive effects on pro-inflammatory cytokine production including IL-1 β, IL-6 and TNF- α (34). Therefore, it is conceivable that the causal effect of *Ruminiclostridium 6* on severe COVID-19 may result from ghrelin.

*Ruminococcus torques*, also known as *Mediterraneibacter torques*, is an anaerobic and gram-positive intestinal bacteria which belongs to the genus *Mediterraneibacter* in the family *Lachnospiraceae*. According to earlier research, *Ruminococcus torques* was positively associated with intestinal paracellular permeability and gastrointestinal disorders (35, 36). Increased intestinal permeability could cause endotoxemia and activate the inflammatory response, which ultimately raises the risk of various diseases including severe illness in COVID-19 patients (37, 38). Additionally, an increase in the abundance of *Ruminococcus torques* is associated with constipation and diarrhoea in children with autism, and the presence of gastrointestinal symptoms has been demonstrated to be an independent risk factor for severe COVID-19 (39, 40). Therefore, *Ruminococcus torques* could have a potential role in the development of severe respiratory symptoms in COVID-19 patients.

Ras interacting protein 1 (RASIP1) is a vascular-specific GTPase signaling regulator involved in a variety of functions, including the Rho signal transmission pathway. RASIP1 regulates the stability of vascular endothelial connections, which is relevant to vascular barrier function, and mediates the regulation of Rho in intrinsic barrier function through Rap1 (41, 42). RASIP1 depletion reduces the barrier function of vascular endothelial cells induced by Rap1 (43). The disruption of endothelium barrier can result in chronic inflammation, atherosclerosis and vascular leakage, as well as the development and progress of COVID-19 (44). Interestingly, *Ruminococcus torques* and RASIP1 were both associated with cell permeability, and in our investigation, they seemed to have a very strong colocalization. Previous database analysis results indicated that rs35866622 decreased the abundance of *Ruminococcus torques* while increased the expression of RASIP1indicating a negative association relationship. As a result, increased abundance of *Ruminococcus torques* coupled with the decreased RASIP1 expression were associated with the disruption of cell barrier and increased permeability, thereby ultimately increase the risk of COVID-19 worsening which was consistent with our MR results.

From the 53 metabolites found to increase the risk of severe COVID-19, we pinpointed the key pathways including ascorbate and aldarate metabolism, beta oxidation of very long chain fatty acids and oxidation of branched chain fatty acids. In these signals, vitamins (ascorbate and aldarate metabolism) have been reported responding to the risk of COVID-19 and its severity. Vitamin C is a potential antiviral agent and may improve immunity. Supplementation with high-dose vitamin C could increase the survival rates of patients with severe COVID-19 by decreasing inflammation and pathogen infectivity and viral yield, improving immune response, alleviating tissue and organ damage (45). Numerous evidences confirm vitamin D insufficiency is associated with greater severity of COVID-19 infection, even the more recent Omicron subvariant of COVID-19 (46–48). Vitamin D administration has been found to be associated with less severe COVID-19 and resulted in a decreased risk of death and admission to intensive care units in patients with COVID-19 (49). In addition, fatty acid metabolism is a crucial event for many viruses to complete their life cycle, and a common consequence of infection by many viruses is to change the nature of lipid metabolism usually from fatty acid oxidation to fatty acid synthesis (50). Fatty acid oxidation is the most powerful pathway to generate energy, and significant impairment in fatty acid oxidation has been reported in patients with post-acute COVID-19 syndrome (51, 52). Our results indicated that the severe COVID-19 causal associated metabolites were significantly enriched in pathways of beta oxidation of very long chain fatty acids and oxidation of branched chain fatty acids. Therefore, fatty acid metabolism offers another promising target to control the COVID-19 infection extent.

It is also worth noting that 5 metabolites (2-tetradecenoyl carnitine, carnitine, cis-4-decenoyl carnitine, decanoylcarnitine and octanoylcarnitine) in the carnitine metabolism pathway were identified to be causal associated with severe COVID-19. Consistent with our findings, a UPLC-MS/MS-based widely targeted metabolomics study also revealed several carnitine family members are significantly reduced in severe COVID-19 patients versus healthy controls subjects and mild COVID-19 patients (53). Carnitine metabolism balance plays an important role in maintaining normal physiological functions through its anti-inflammatory, antioxidative, anti-apoptotic, anti-fibrosis and biomembrane-stabilizing properties (54). Carnitine deficiency occurs in multiple diseases such as sepsis, advanced liver cirrhosis and endocrine disorders (54). Severe COVID-19 patients usually exhibit metabolic disorders and multiple organ dysfunctions, the downregulated carnitine in the severe patients may contribute to impaired organ function. Additionally, alanine, as another important metabolites for COVID-19 severity, was revealed by the 4 identified metabolites (aspartylphenylalanine, leucylalanine (X-14189), leucylalanine (X-14304) and N-acetylalanine). A key physiological function of alanine is to transport pyruvate and glutamate from the muscles to the liver, a process known as the glucose–alanine cycle. Data from patients with different severity grades of COVID-19 show that circulating pyruvate level was the strongest determinants of severe COVID-19 (55), and a meta-analysis indicated that elevated glutamate was associated with an increased risk of COVID-19 severity (56).

In this study, we set 131 taxa and 452 metabolites as exposure factors, severe COVID-19 as the outcome variable, and then grouped the COVID-19 cohort according to the IVs selected for each exposure factor. Then, by analyzing the differences in the risk of severe COVID-19 between the exposed and control groups, we could infer the causal relationship between exposure factors and severe COVID-19. Our study showed that MR analysis could sort out some intestinal bacteria and metabolites that have a potential relationship with COVID-19 severity. In addition, by combining two-stage MR analysis and mediation analysis, we successfully linked gut microbiome and plasma metabolome, and constructed some pathways from intestinal bacteria to severe COVID-19 through plasma metabolites or from plasma metabolites to severe COVID-19 through intestinal bacteria. Our study provides potential biomarkers associated with severe COVID-19 and can benefit the mechanistic investigation of severe COVID-19.

This study has some limitations. Firstly, our casual inference results of intestinal bacteria might be affected by confounding factors such as race, diet, and disease status. Additionally, no experiments have been conducted to validate the causal associations of this study. Therefore, mechanistic studies will be necessary in the future to verify these relationships.

## Conclusion

In conclusion, our comprehensive MR analyses identified 13 human intestinal taxa and 53 human serum metabolites potential causal associated with COVID-19 severity. We also found 11 mediated relations among the identified intestinal taxa and serum metabolites. These causal taxa and metabolites potentially served as clinical biomarkers for risk stratification and prognostication of severe COVID-19 and would benefit the mechanism mechanistic investigation of severe COVID-19.

## Data availability statement

The raw data supporting the conclusions of this article will be made available by the authors, without undue reservation.

## Ethics statement

The study was approved by the ethics committee of Institute of Clinical Pharmacology, Central South University.

## Author contributions

XL, LZ and J-QL participated in research design. HY, LZ, J-QL, SZ and WZ participated in the writing of the paper. H-XH, PX, X-HC and Y-DQ participated in the performance of the research. XL and HY prepared the figures. All authors contributed to the article and approved the submitted version.
